# AI-Based Detection of Oral Squamous Cell Carcinoma with Raman Histology

**DOI:** 10.3390/cancers16040689

**Published:** 2024-02-06

**Authors:** Andreas Weber, Kathrin Enderle-Ammour, Konrad Kurowski, Marc C. Metzger, Philipp Poxleitner, Martin Werner, René Rothweiler, Jürgen Beck, Jakob Straehle, Rainer Schmelzeisen, David Steybe, Peter Bronsert

**Affiliations:** 1 Institute for Surgical Pathology, Medical Center, University of Freiburg, 79106 Freiburg, Germany; 2 Faculty of Biology, University of Freiburg, 79104 Freiburg, Germany; 3 Tumorbank Comprehensive Cancer Center Freiburg, Medical Center, University of Freiburg, 79106 Freiburg, Germany; 4 Core Facility for Histopathology and Digital Pathology, Medical Center, University of Freiburg, 79106 Freiburg, Germany; 5 Department of Oral and Maxillofacial Surgery, Medical Center, University of Freiburg, 79106 Freiburg, Germany; 6 Center for Advanced Surgical Tissue Analysis (CAST), University of Freiburg, 79106 Freiburg, Germany; 7 Department of Oral and Maxillofacial Surgery and Facial Plastic Surgery, University Hospital, LMU Munich, 80337 Munich, Germany; 8 Department of Neurosurgery, Medical Center, University of Freiburg, 79106 Freiburg, Germany

**Keywords:** machine learning, neural networks, pathology, computational biology, head and neck neoplasms

## Abstract

**Simple Summary:**

Stimulated Raman Histology (SRH) is a technique that uses laser light to create detailed images of tissues without the need for traditional staining. This study aimed to use deep learning to classify oral squamous cell carcinoma (OSCC) and different non-malignant tissue types using SRH images. The performances of the classifications between SRH images and the original images obtained from stimulated Raman scattering (SRS) were compared. A deep learning model was trained on 64 images and tested on 16, showing that it could effectively identify tissue types during surgery, potentially speeding up decision making in oral cancer surgery.

**Abstract:**

Stimulated Raman Histology (SRH) employs the stimulated Raman scattering (SRS) of photons at biomolecules in tissue samples to generate histological images. Subsequent pathological analysis allows for an intraoperative evaluation without the need for sectioning and staining. The objective of this study was to investigate a deep learning-based classification of oral squamous cell carcinoma (OSCC) and the sub-classification of non-malignant tissue types, as well as to compare the performances of the classifier between SRS and SRH images. Raman shifts were measured at wavenumbers k_1_ = 2845 cm^−1^ and k_2_ = 2930 cm^−1^. SRS images were transformed into SRH images resembling traditional H&E-stained frozen sections. The annotation of 6 tissue types was performed on images obtained from 80 tissue samples from eight OSCC patients. A VGG19-based convolutional neural network was then trained on 64 SRS images (and corresponding SRH images) and tested on 16. A balanced accuracy of 0.90 (0.87 for SRH images) and F1-scores of 0.91 (0.91 for SRH) for stroma, 0.98 (0.96 for SRH) for adipose tissue, 0.90 (0.87 for SRH) for squamous epithelium, 0.92 (0.76 for SRH) for muscle, 0.87 (0.90 for SRH) for glandular tissue, and 0.88 (0.87 for SRH) for tumor were achieved. The results of this study demonstrate the suitability of deep learning for the intraoperative identification of tissue types directly on SRS and SRH images.

## 1. Introduction

Due to their significant influence on recurrence-free survival, the assessments of surgical margins play a pivotal role in the operative treatment of oral squamous cell carcinoma (OSCC) [1,2,3,4]. The conventional approach for the intraoperative evaluation of resection margins is based on the preparation of hematoxylin and eosin (H&E)-stained frozen sections [5]. To generate these sections, tissue samples are frozen, sectioned into thin slices, stained, and then microscopically examined by a board-certified pathologist. This technique provides intraoperative real-time feedback on the resection status, enabling surgeons to extend the resection in case of tumor-positive margins [6].

Stimulated Raman Histology (SRH) [7,8] addresses challenges associated with conventional frozen section analysis, which utilizes fiber laser-based Stimulated Raman Scattering (SRS) microscopy to generate images resembling the appearance of H&E-stained tissue sections from fresh tissue specimens without the need for preprocessing. The NIO Laser Imaging System (Invenio Imaging Inc., Santa Clara, CA, USA) is a movable, standalone clinical SRS microscope that facilitates the application of this technique in an intraoperative setting. It measures energy shifts via Raman scattering [9] at wavenumbers of k_1_ = 2845 cm^−1^ and k_2_ = 2930 cm^−1^. As photons with a wavenumber of k_1_ scatter mostly at CH_2_ bonds, abundant in lipids, and photons with a wavenumber of k_2_ scatter at CH_3_ bonds, predominant in proteins and DNA, these spectral data depict the spatial distribution of lipids, proteins, and DNA in the tissue sections. To facilitate visual assessment, the data are subsequently subjected to a coloring algorithm included in the NIO Laser Imaging System software version 1.6.0, providing images similar to conventional H&E-stained sections.

In recent years, the field of medicine has witnessed a paradigm shift with the advent of deep learning (DL) applications. Deep learning is a subfield of machine learning that involves the use of artificial neural networks composed of multiple layers of interconnected nodes that are capable of representing complex patterns in data and automatically make predictions without explicit programming [10]. DL has revolutionized various aspects of medicine such as radiology [11], neurology [12], and cardiology [13]. As a promising technique, DL has found its way into the evaluation of histopathological images [14,15,16]. In this context, DL-based algorithms have exhibited promising outcomes in assessing various tissue and tumor types, including OSCC, in conventional histopathology sections [17]. In the domain of SRH, encouraging results have already been reported when applying this approach to the evaluation of SRH images obtained from brain tumor tissue [18]. Considering the potential of SRH in the assessment of tissue sections from patients with OSCC and the possibilities of DL-driven image evaluations, the combination of these two approaches could offer promising options in an accelerated intraoperative assessment of tissue samples from OSCC patients.

Thus, the objective of the present study was to assess a DL-based approach for the identification of OSCC in SRS and SRH images and for the further sub-classification of non-neoplastic tissues.

## 2. Materials and Methods

### 2.1. Study Protocol and Sample Acquisition

This prospective study received ethical approval from the Ethics Committee of the University of Freiburg (Reference: #22-1037). Prior to inclusion into the study, informed written consent was obtained from all patients. The inclusion criteria comprised individuals of legal age (>18 years) with biopsy-confirmed OSCC, without prior neoadjuvant therapy and with an indication for surgical resection. In total, 80 tissue specimens were collected from 8 patients as part of this prospective study during the period of May to July 2022.

### 2.2. SRH Image Acquisition

The acquisition of SRH images was performed as previously reported [19]. In brief, native tissue samples, each with a maximum size of 0.4 × 0.4 × 0.2 cm, were extracted from areas macroscopically suspected of tumor presence, as well as from areas that appeared macroscopically non-neoplastic. The selection of areas suspected of tumor presence was guided by morphologic criteria such as ulcerative and exophytic alterations. The rationale behind this procedure was to achieve a balanced distribution of tumor-positive and tumor-free tissue samples for subsequent analyses. Each tissue specimen was positioned on a custom microscope slide. The resulting SRS images portray the spatial distribution of the CH_2_ and CH_3_ bonds across the tissue samples via Raman scattering. These images were obtained through multiple line scans, each spanning a width of 1000 pixels (with a pixel size of 467 nm) and were taken at a depth of 10 µm below the coverslip. The pixel size and scanning depth are predetermined settings in the NIO Laser Imaging System. With the scanning time increasing as the pixel size decreases, 467 nm was the preset value chosen to strike a favorable balance between scanning time and image resolution. In terms of scanning depth, the objective was to reduce image noise while simultaneously addressing surface irregularities. In this context, a scanning depth of 10 μm was identified as providing a favorable tradeoff. In the workflow of the NIO Laser Imaging System, the two-channel SRS images were further converted with a special look-up table to provide images reminiscent of conventional H&E-stained slides. Hereinafter, we will refer to images showing the energy shift at the two aforementioned wavenumbers as SRS images, and to images generated with the vendor-specific look-up table, resembling H&E-stained images, as SRH images.

### 2.3. Histopathological Evaluation

**Figure 1:**
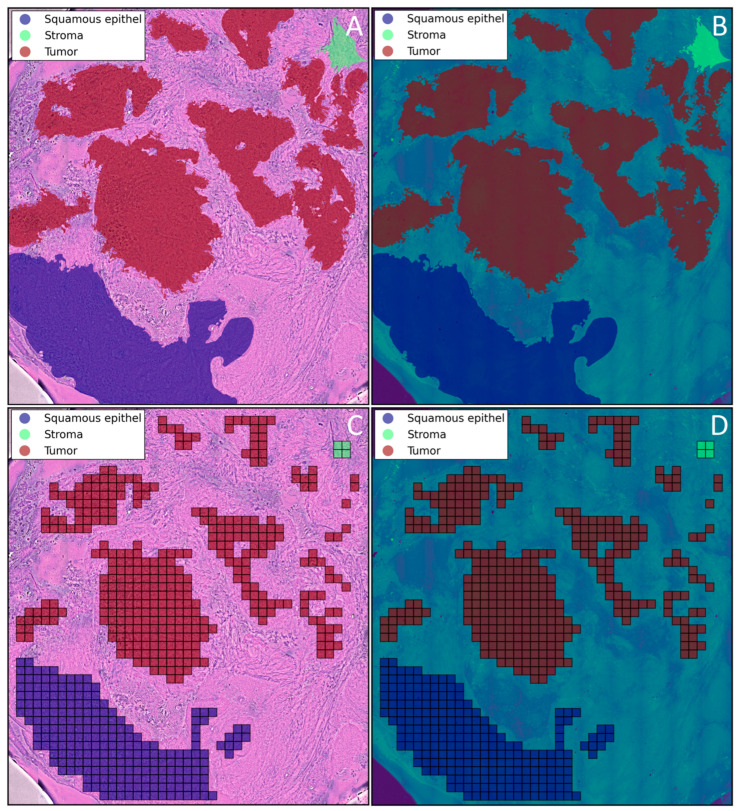
Annotations of tissue classes “Squamous epithelium”, “Stroma”, and “Tumor” on an SRH image (**A**) and transferred annotations on a corresponding SRS image (**B**) as well as tiles generated from the annotations with class labels “Squamous epithelium”, “Stroma”, and “Tumor” on a SRH image (**C**) and on the corresponding SRS image (**D**). Only tiles that intersect with an annotation by 99% were kept for the generation of the dataset.

In total, 80 SRH images were obtained from 8 patients diagnosed with OSCC. All SRH images were imported into QuPath Version v0.4.3 [20], and the regions of interest within these images were categorized into one of the following six groups: tumor, stroma, adipose tissue, muscle, squamous epithelium, and glandular tissue. Experienced pathologists performed annotations on all 80 images using the wand and brush tools, as well as manual annotations. Annotations were conducted only if the tissue could be clearly classified into one of the aforementioned groups. A total of 877 annotations were made across all 80 specimens, ranging from 1 to 52 annotations per specimen and varying in size. The annotations were exported from QuPath as GeoJSON files for further usage. Notably, since the SRH images were directly generated from the SRS images, the annotations made on the SRH images could be seamlessly transferred to the corresponding SRS images (see Figure 1).

### 2.4. Generation of the Dataset

The SRS images are pixel arrays with two channels, one for storing the scattering values of each of the two wavenumbers. Since DL algorithms suitable for image processing mainly require images with three channels, we populated the first two channels of an empty array with scattering values representing the CH_2_ and CH_3_ bonds. The third channel of the array was populated with the spectral difference CH_3_-CH_2_ for each pixel following the approach of [18]. Subsequently, all labeled SRS and SRH images were divided into tiles measuring 250 × 250 pixels. A tile was considered labeled if at least 99% of its area overlapped with an annotated region; tiles that did not meet this criterion were excluded from the dataset. A threshold of 99% ensures that tiles contain almost only labeled pixels. A lower threshold would include more unlabeled pixels (and therefore non-relevant) or even contradictory information about the label into the respective tile. In effect, this would increase the difficulty of the prediction task for the neural network. Additionally, tiles at the periphery of an annotation where some pixels at a certain edge of the tile are not within the annotation were considered to include sufficient labeled information. The final dataset comprised 21,703 tiles, distributed as follows: 4892 tumor, 4902 stroma, 1471 adipose tissue, 756 muscle, 8461 squamous epithelium, and 1221 glandular tissue tiles.

### 2.5. Data Split and Class Distribution

The dataset was divided into a training set comprising 64 images (80% of the dataset) and a test set consisting of 16 images (20% of the dataset). Within the training set, 10% was allocated as a validation set. Table 1 provides an overview of the relative class distribution of the training, validation, and test sets, as well as the entire dataset. Achieving an identical class distribution for all subsets was not possible due to variations in class abundance across different images. To approximate a similar class distribution among all subsets, we iteratively computed the Jensen–Shannon Distance between the class distributions of the subsets. During each iteration, random images from the dataset were selected and added to a subset. An image remained in the respective subset if the Jensen–Shannon Distance between the class distribution of the subset and that of the whole dataset decreased; otherwise, it was discarded, and the next image was considered. This iterative process continued until the desired number of images was included in each subset, with the boundary condition that every class had to be present in each of the subsets.

### 2.6. Deep Learning-Based Evaluation of Images

A convolutional neural network (CNN) based on the architecture of VGG19 [21] with randomly initialized weights was used. The choice of a VGG19 architecture was based on the fact that it outperformed other architectures like GoogLeNet [22] or ResNet50 [23] on histology slides in the study presented by Kather et al. [14]. The input dimension of the CNN was (batch size, 250, 250, 3), and the output dimension was (batch size, 6). To account for rotational invariance, all tiles were randomly flipped horizontally and vertically before being fed to the neural network. Two fully connected layers were added to the end of the CNN with 1000 and 100 neurons, respectively. A dropout layer was inserted between the last two fully connected layers with a dropout probability of 0.5 active during training. The CNN was trained for 100 epochs with a batch size of 100 and a learning rate of 0.0001. All computations were performed with Python 3.9.16 and Tensorflow 2.6.0 [24] on a NVIDIA Geforce RTX 4090. Class imbalance was addressed by weighting the loss function during training according to the overall class distribution.

### 2.7. Statistical Evaluation

The statistical evaluation of the CNN performance was conducted using the metrics of precision, recall, and F1-score for each class. Precision is defined as the number of true positives divided by the sum of true positives and false positives, representing the proportion of tiles predicted to belong to a certain class that actually belong to that class. Recall is defined as the number of true positives divided by the sum of true positives and false negatives, representing the proportion of tiles of a certain class correctly predicted. The F1-score, which is the harmonic mean of precision and recall [25], is defined as follows:

$$F1=2\times \frac{precision\times recall}{precision+recall}$$

All metrics have a value range in the closed interval between 0 and 1, where 1 indicates the best performance, and 0 the worst. To assess the overall performance across all classes, we report the balanced accuracy score, which accounts for imbalanced datasets and is the average of the recall scores per class. Additionally, the confusion matrix illustrates the number of tiles predicted to belong to a certain class over the true classes for each class of the test set.

## 3. Results

The neural network demonstrated the ability to detect different tissue types on SRS images (and corresponding SRH images) with an overall balanced accuracy of 0.90 (0.87). Stromal tissue tiles exhibited a precision of 0.90 (0.90) and a recall of 0.91 (0.92). Adipose tissue tiles displayed a precision of 0.97 (0.99) and a recall of 0.98 (0.94). The performance for squamous epithelium, while slightly lower, still achieved a precision of 0.89 (0.82) and a recall of 0.90 (0.84). Tiles belonging to the muscle class were identified with a precision of 0.95 (0.79) and a recall of 0.89 (0.73). For glandular tissue, the precision was 0.92 (0.96), with a recall of 0.82 (0.85). The identification of squamous cell carcinoma resulted in a precision of 0.86 (0.92) and a recall of 0.90 (0.82), leading to an F1-score of 0.88 (0.87).

**Figure 2:**
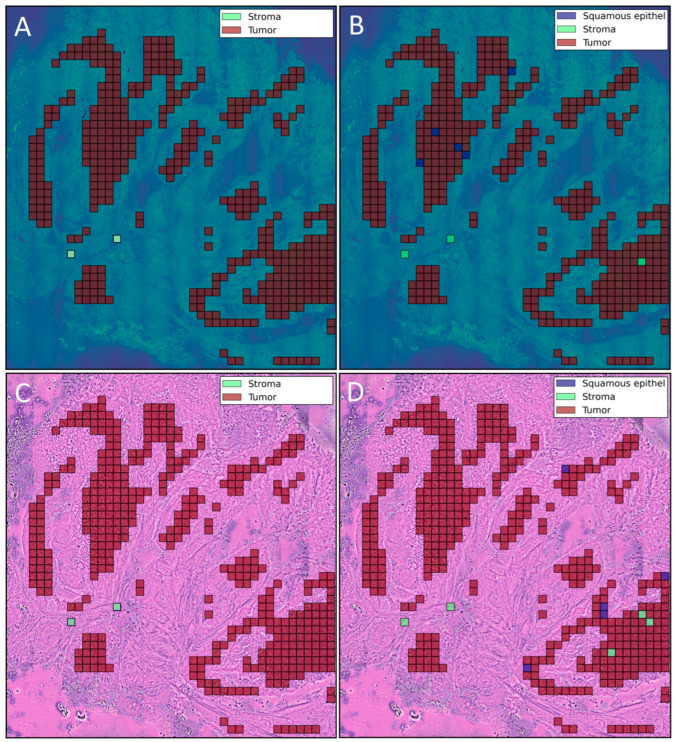
Ground truth class labels for each tile (**A**) and predicted class labels for each tile (**B**) on a sample SRS image. Both true tiles with class label “Stroma” were classified correctly, whereas 6 tiles with class label “Tumor” were incorrectly classified as “Squamous epithelium” (5 tiles) and “Stroma” (1 tile). Ground truth class labels for each tile (**C**) and predicted class labels for each tile (**D**) on a sample SRH image. Both true tiles with class label “Stroma” were classified correctly, whereas 8 tiles with class label “Tumor” were incorrectly classified as “Squamous epithelium” (5 tiles) and “Stroma” (3 tiles).

An example of ground truth class labels for each tile and corresponding predictions for SRS and SRH image can be seen in Figure 2. For stroma tissue, the metrics reveal a better performance of the neural network on the SRH images compared to SRS images, and for glandular tissue, a similar performance was observed. However, an overall comparison of the balanced accuracy reveals a slight outperformance of the neural network on the SRS images compared to the performance on the SRH images. Regarding the detection of OSCC, the performances on both data modalities show similar results.

**Figure 3:**
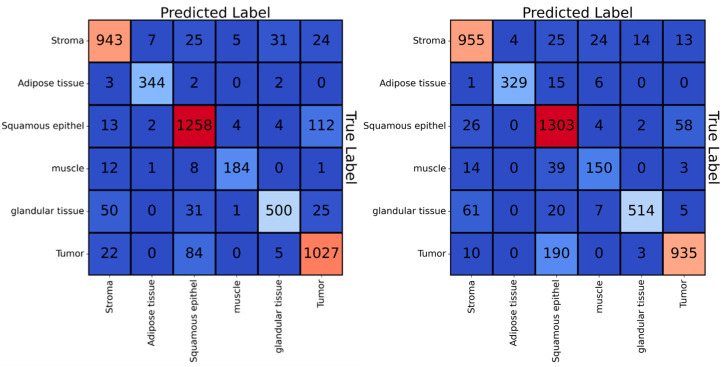
Confusion matrices for the classification of the CNN on the SRS test dataset (**left**) and the corresponding SRH test dataset (**right**). The diverging colormap shows small values in dark blue with increasing brightness according to increasing values. Large values are shown in dark red with decreasing brightness according to increasing values.

The dataset’s class imbalance appears to have been effectively addressed by the weighting of the loss function during training, as underrepresented classes did not exhibit worse performances than overrepresented ones. Detailed performance metrics for all classes on the SRS images (and SRH images, respectively) can be found in Table 2. Figure 3 illustrates the confusion matrices, displaying the number of predicted and true labels for each tile in the test.

For both SRS and SRH images, the confusion matrices highlight that the primary source of error lies in the confusion between squamous epithelium and tumor. Specifically, for SRS images, the neural network misclassified 8% (112 out of 1393) of squamous tissue tiles as tumor and 7% (85 out of 1138) of tumor tiles as squamous tissue. For SRH images, the neural network misclassified 4% (58 out of 1393) of squamous tissue tiles as tumor tiles and 17% (190 out of 1138) of tumor tiles as squamous tissue tiles.

## 4. Discussion

This study aimed to assess the performance of a DL-based identification of oral squamous cell carcinoma and the subclassification of non-malignant tissue on SRS and SRH images. SRH is a novel technology that provides images reminiscent of conventional H&E-stained slides without the need for sectioning and staining. By leveraging the inherently digital nature of these images, DL-based evaluation has the potential to expedite diagnoses and facilitate intraoperative surgical decision making.

Deep learning applications in pathology have made significant advancements in recent years, encompassing various cancer types and supporting diagnosis, classification, grading, and staging methods [26,27,28,29]. In the field of pathohistological diagnosis, promising results have emerged for the automated detection of numerous cancers in whole-slide images of conventional histology slides. This includes OSCC, where current investigations have reported F1-scores > 0.90 [17,30].

In the present study, a convolutional neural network based on the VGG19 architecture [21] with randomly initialized weights was used. The CNN was trained to assign one of six class labels to tiles of SRS and SRH images. The performance on the hold-out test set demonstrates the neural network’s ability to identify OSCC and to subclassify non-malignant tissue types such as stroma, adipose tissue, squamous epithelium, muscle, and glandular tissue. The CNN’s performance varied across tissue types, with the highest performance observed for adipose tissue (F1-score of 0.98 on SRS images and 0.96 on SRH images), and the lowest for glandular tissue (F1-score of 0.87 on SRS images) and muscle tissue (F1-score of 0.76 on SRH images). Possible explanations for this finding could be the variations in tissue composition, heterogeneity of tissue structure, and the fact that certain tissue types are represented more characteristically in SRH than others (e.g., the characteristic representation of adipose tissue with deep-purple vacuoles).

Regarding the confusion between squamous epithelium and tumor, it must be considered that normal mucosa can be difficult to distinguish from carcinoma cells, especially from precursor lesions at the cellular level in SRH morphology.

While this study represents the first exploration of a DL-based evaluation of SRH images from OSCC patients, results are available from previous investigations in the field of neurosurgery, where SRH originated [18]. These findings include the use of patch-level CNN predictions for glioma recurrence detection, achieving a diagnostic accuracy of 95.8% and the use of the Inception-ResNet-v2 architecture for detecting common central nervous system tumors, with a CNN-based diagnosis of SRH images shown to be non-inferior to a pathologist-based interpretation of conventional histologic images (overall accuracy, 94.6% vs. 93.9%). Additionally, the automated analysis of skull base tumor specimens for the detection of various tumor types has applied ResNet architectures that resulted in overall diagnostic accuracies of 91.5% with cross-entropy, 83.9% with self-supervised contrastive learning, and 96.6% with supervised contrastive learning.

The CNN-based prediction of brain tumor diagnosis reported by [18] yielded a mean class accuracy of 89.2% (at the patient level), which is comparable to the mean class accuracy (equivalent to the mean of the recall values) of 90.0% for SRS images (and 86.7% for SRH images) presented in our study. The authors of [18] predicted 13 class labels using a training dataset comprising over 2.5 million labeled tiles from 415 patients. In contrast, our study predicted six class labels with a training dataset comprising a number of labeled tiles several orders of magnitude lower (16,973). Considering the relatively low number of tiles in the training set, the performance of the CNN is remarkable. Factors that complicate a direct comparison between the results reported by [18] and our study include differences in CNN architecture (Inception-ResNet-v2 in [18] vs. VGG19-based in our study), the covered inter-patient variety of the dataset (415 patients in [18] vs. 8 patients in our study), and the disease-specific complexity of the mapping between morphology and histological classification (brain tumor in [18] vs. OSCC in our study).

While the present study yielded promising results, the limitations of this method could include its use on rare tumor entities, tumor precursors, or distinctly large inflammatory reactive changes. Further the training of DL algorithms is needed for tumor entities not included in the study so far, such as rare subtypes of adenocarcinomas or hematological illnesses or lymphomas.

To enhance the neural network’s performance, larger and more diverse datasets are imperative. On the algorithmic front, aggregating predictions from multiple neural networks through ensemble learning could lead to increased stability and performance improvements. However, high throughput is not yet possible due to the described restrictions and monitoring.

A clear advantage of SRH is that, in contrast to H&E and other routinely used staining procedures and the associated laboratory-triggered deviations, the technique, and thus the image to be assessed, do not differ. This can provide internationally standardized, homogeneous modularity. In the future, a globally accessible open-source database with scattering spectra for comparable histology is conceivable.

For a DL-based classifier to be effectively deployed in clinical practice, it must not only demonstrate high performance metrics but also exhibit stability across diverse datasets and remain resilient to performance errors caused by slight variations in input data. Moreover, issues of trustworthiness and transparency arise, especially in the medical field, where incorrect predictions can lead to erroneous diagnoses with potentially severe consequences for patients.

## 5. Conclusions

In recent years, there has been a concerted effort to digitize and automate histopathological workflows. Within this context, Raman scattering has emerged as a promising technology, and the inherently digital format of images obtained from Stimulated Raman Scattering and Stimulated Raman Histology provides an ideal foundation for deep learning-based image analyses. The results of this study illustrate the significant potential of integrating SRH and deep learning to advance the digitization of workflows in the surgical treatment of oral squamous cell carcinoma.

## Figures and Tables

**Table 1:** Relative class distributions for the entire dataset (total), training set, validation set, and test set.

	Tumor	Stroma	Adipose Tissue	Muscle	Squamous Epithelium	Glandular Tissue
Total	0.23	0.23	0.07	0.03	0.39	0.05
Training set	0.25	0.26	0.06	0.03	0.37	0.03
Validation Set	0.31	0.28	0.17	0.03	0.16	0.05
Test Set	0.24	0.22	0.07	0.04	0.30	0.13

**Table 2:** Performance metrics of the CNN on the test set on SRS images (SRH images) as well as the number of tiles for each class in the test set.

	Precision	Recall	F1-Score	Number of Tiles
Stroma	0.90 (0.90)	0.91 (0.92)	0.91 (0.91)	1035
Adipose tissue	0.97 (0.99)	0.98 (0.94)	0.98 (0.96)	351
Squamous epithelium	0.89 (0.82)	0.90 (0.94)	0.90 (0.87)	1393
Muscle	0.95 (0.79)	0.89 (0.73)	0.92 (0.76)	206
Glandular tissue	0.92 (0.96)	0.82 (0.85)	0.87 (0.90)	607
Tumor	0.86 (0.92)	0.90 (0.82)	0.88 (0.87)	1138

## Data Availability

Data supporting the findings of the study are available from the corresponding author upon reasonable request.
